# Light triggered release of a triple action porphyrin-cisplatin conjugate evokes stronger immunogenic cell death for chemotherapy, photodynamic therapy and cancer immunotherapy

**DOI:** 10.1186/s12951-022-01531-5

**Published:** 2022-07-16

**Authors:** Haiqin Song, Zhenghao Cai, Juyi Li, Haihua Xiao, Ruogu Qi, Minhua Zheng

**Affiliations:** 1 Department of General Surgery, School of Medicine, Ruijin Hospital, Shanghai Jiaotong University, Shanghai, 20023 China; 2 Department of Materials Science and Chemical Engineering, Stony Brook University, Stony Brook, NY 11794 USA; 3 Beijing National Laboratory for Molecular Sciences, State Key Laboratory of Polymer Physics and Chemistry, Institute of Chemistry, Chinese Academy of Sciences, Beijing, 100190 China; 4 School of Medicine and Holistic Integrative Medicine, Nanjing University of Chinese Medicine, Nanjing, 210023 China

**Keywords:** Porphyrin, Cisplatin, Immunogenic cell death, Cancer immunotherapy, Photodynamic therapy, Nanoparticles

## Abstract

Photodynamic therapy (PDT) has emerged as an attractive therapeutic approach which can elicit immunogenic cell death (ICD). However, current ICD inducers are still very limited as the representative ICD induces of photosensitizers can only evoke insufficient ICD to achieve unsatisfactory cancer immunotherapy. Herein, we demonstrated the use of a triple action cationic porphyrin-cisplatin conjugate (Pt-1) for drug delivery by a reactive oxygen species (ROS) sensitive polymer as nanoparticles (NP@Pt-1) for combined chemotherapy, PDT and immunotherapy. This unique triple action Pt-1 contains both chemotherapeutic Pt drugs and Porphyrin as a photosensitizer to generate ROS for PDT. Moreover, the ROS generated by Pt-1 can on the one hand degrade polymer carriers to release Pt-1 for chemotherapy and PDT. On the other hand, the ROS generated by Pt-1 subsequently triggered the ICD cascade for immunotherapy. Taken together, we demonstrated that NP@Pt-1 were the most effective and worked in a triple way. This study could provide us with new insight into the development of nanomedicine for chemotherapy, PDT as well as cancer immunotherapy.

**Supplementary Information:**

The online version contains supplementary material available at 10.1186/s12951-022-01531-5.

## Introduction

Immunogenic cell death (ICD), characterized by the antigenicity and adjuvanticity of dying cancerous cells, has emerged as an alternative approach by stimulating innate and adaptive immune responses with activated antitumor immunity to induce the tumor inhibition and generation of long-term immunological memory [1–3]. When tumor cells undergoes the so-called ICD effect, a series of signal molecules and cytokines are involved, including the expression of signal molecules on the cell membrane surface and the production and release of pro-immune effectors which are called damage associated molecular patterns (DAMPs), including the release of over-expressed calreticulin (CRT) and adenosine triphosphate (ATP), high mobility group protein B1 (HMGB1), so as to enhance the immunogenicity of tumor cells, recruit dendritic cells (DC) to the tumor sites, and activate specific cytotoxic T lymphocytes (CTL) to attack the tumor cells. ICD effect was proved to be able to improve current cancer treatment [4–6]. By monitoring the changes of tumor cell immunogenicity before and after chemotherapy and combining chemotherapy and immunotherapy, the therapeutic effect of tumor could be greatly enhanced [7, 8].

A variety of anticancer treatment modalities may produce ICD effect, including chemotherapeutic agents (oxaliplatin, doxorubicin, and camptothecin), radiotherapy, and photodynamic therapy (PDT) [9–11]. Among them, PDT is beneficial which utilizes photosensitizer to produce reactive oxygen species (ROS), such as singlet oxygen (^1^O_2_) under suitable light irradiation, to induce intracellular stress and initiate the release of DAMPs to trigger ICD cascade [12, 13]. Despite of the promising approaches via ICD effect for cancer immunotherapy, most photosensitizers also shows marginal to moderate therapeutic performance with relatively minimal side effects and improved tumor specific killing compared with traditional chemotherapy [14, 15]. This might be due to the fact that current photosensitizers mostly work in a single way which are unable to elicit sufficient ICD effect. The combination of chemotherapy and PDT may be helpful to solve this problem for effectively improving cancer immunotherapy by maximumly inducing the ICD cascade [16]. However, currently, the clinical translation of chemotherapeutic drugs and PDT-based photosensitizers for ICD was hampered due to the great toxicity, short blood circulation, limited intratumoral accumulation and retention, and minimized tumor cellular uptake of these agents [17].

To address these challenges, nanotechnology is applied to offer great opportunities to overcome these drawbacks [18, 19]. The tunable morphology and variable surface properties of nanoparticles enable specific payload accumulation at the tumor site, enhancing the tissue penetration and increasing cellular uptake, as well as the controlled release of ICD inducers [20]. Moreover, the remarkable development of internal tumor microenvironment (TME) responsive (such as pH, intracellular glutathione/reactive oxygen species, enzyme) and external stimuli-responsive (such as light, ultrasound, and heat, etc.) delivery systems have been extensively explored to optimize the cancer immunotherapy [21–23]. Taken together, nanoparticle-based delivery system has attracted increasing attention as an applicable anticancer immunotherapy platform.

**Scheme 1:**
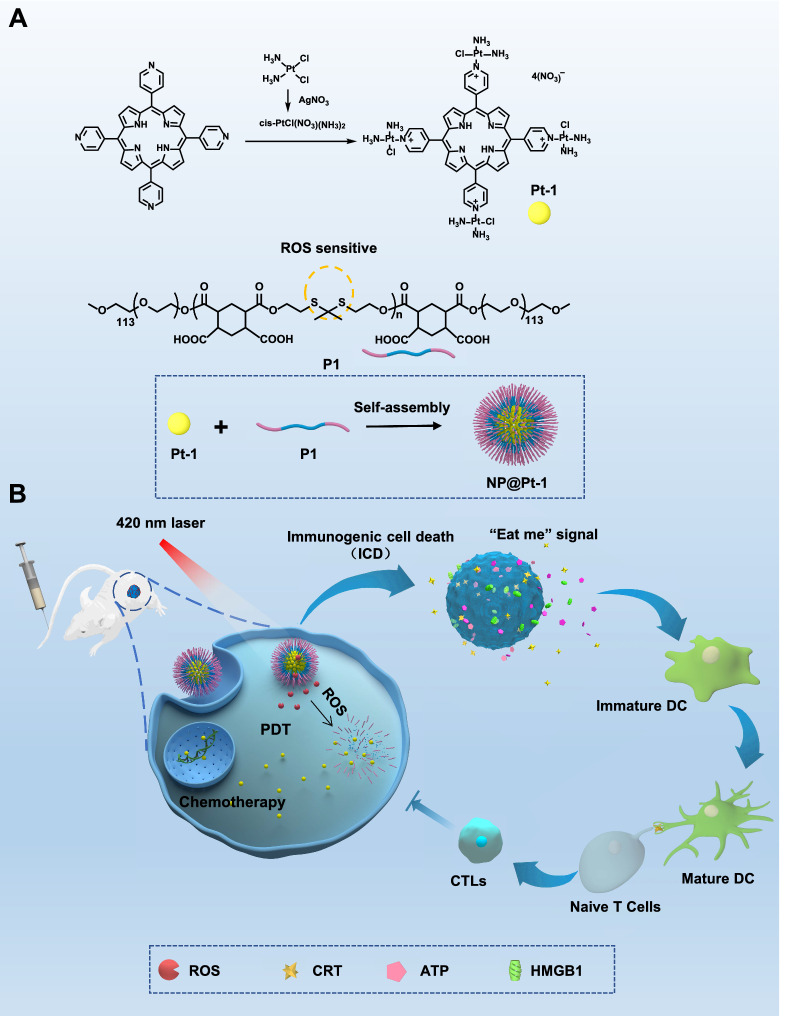
Schematic illustration of the triple action NP@Pt-1 for chemotherapy, PDT and cancer immunotherapy. **A** Chemical structures of Pt-1 and the ROS sensitive polymer carrier (P1) and their assembly into NP@Pt-1. **B** Illustration of the triple action of NP@Pt-1. NP@Pt-1 were injected to CT-26 tumor bearing mice and were then accumulated to the tumor site. Under light irradiation (420 nm), numerous ROS was generated to break down the polymer chain (thioketal bonds), resulting in the release of Pt-1. Pt-1 then chelated with intracellular DNA to induce apoptosis for chemotherapy. Moreover, the ROS can on the one hand damage the cell membranes, proteins and DNAs in cancer cells for PDT. On the other hand, it can induce ICD effect by sending out “eat me” signal for cancer immunotherapy

Herein, we rationally designed a unique triple action cationic porphyrin-cisplatin conjugate (Pt-1, Scheme 1). The advantage for the design of Pt-1 is that on the one hand the porphyrin as its core can generate ROS for PDT as well as ICD effect for cancer immunotherapy under light irritation. On the other hand, as a cisplatin-like molecule, Pt-1 can interact with intracellular DNA for Pt–DNA binding to induce cell apoptosis for chemotherapy. In this way, Pt-1 works in a concerted way in a triple mode for cancer therapy. Moreover, the cationic Pt-1 can self-assemble into nanoparticle in the presence of an anionic ROS sensitive biodegradable polymer with numerous thioketal bonds in the polymer main chain and pendant carboxylic acids (P1, Scheme 1) via electronic interactions (NP@Pt-1, Scheme 1A). We hypothesized that the combination of PDT and chemotherapy of NP@Pt-1 could increase the anticancer efficacy, induce intracellular oxidative stress and elicit a greater ICD effect. To further validate our hypothesis, a murine colon cancer (CT26) model was established to evaluate the therapeutic efficacy of NP@Pt-1 in vivo (Scheme 1B). We found that NP@Pt-1 could be accumulated at the tumor sites and were taken up into the tumor cells via endocytosis after intravenous injection (i.v.). Subsequent local irradiation by 420 nm light at the tumor site could: (1) induce ROS generation from porphyrin of Pt-1 for PDT and simultaneously promote the degradation of the polymer main chain to dissociate the nanoparticles [24, 25]; (2) boost the chelation of platinum to intracellular DNA thereby inducing apoptosis for chemotherapy; (3) induce the ICD cascade which could further trigger the DAMPs released from dying cells to promote the maturation of DC and activation of toxic T cells for cancer immunotherapy (Scheme 1B). In this way, NP@Pt-1 worked in a concerted triple way for chemotherapy, PDT, and cancer immunotherapy.

## Results and discussion

**Fig. 1:**
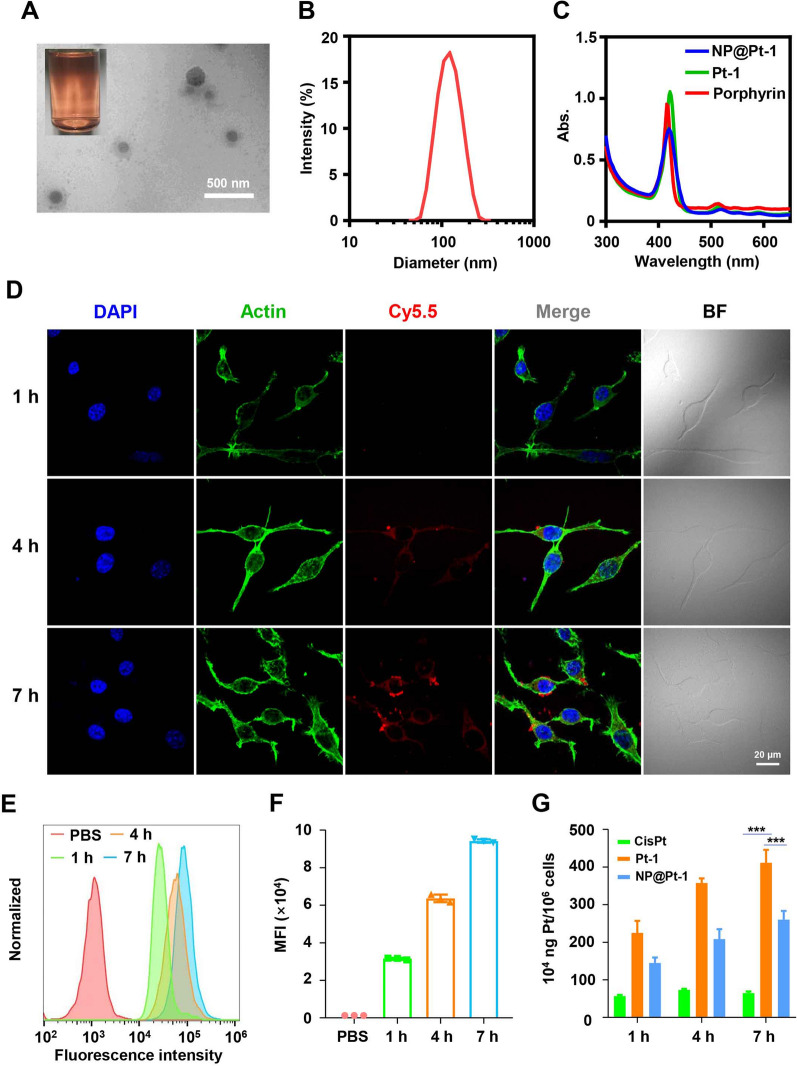
Characterization and intracellular uptake of NP@Pt-1. **A** Photograph images of aqueous solution of NP@Pt-1 and TEM images of NP@Pt-1. Scale bar: 500 nm. **B** Diameter of NP@Pt-1 in water. **C** UV–vis spectra of porphyrin, Pt-1, and NP@Pt-1. **D** CLSM images of CT26 murine colon cancer cells treated with Cy5.5 labeled NP@Pt-1 at 1 h, 4 h, and 7 h respectively. Scale bar = 20 μm. **E** Endocytosis of Cy5.5 labeled NP@Pt-1 by CT26 cells via flow cytometry at 1 h, 4 h and 7 h respectively. **F** Quantification of the intracellular uptake of Cy5.5 labeled NP@Pt-1 in (**E**). **G** ICP-MS quantification of intracellular Pt uptake by CT26 cells exposed to various Pt-containing drugs treatment. (n = 3, ***p < 0.001)

Generally, Pt-1 was prepared by simply mixing cisplatin and porphyrin in two days [26]. The as-prepared cationic Pt-1 owns one porphyrin core for ROS generation and PDT as well as four cisplatin-like units for chemotherapy. The successful synthesis of Pt-1 was confirmed by ^1^H NMR (Additional file 1: Fig. S1–S3). To promote drug loading and intracellular-triggered release of Pt-1, P1 was designed with ROS sensitive thioketal linkages in the polymer main chain [27]. Subsequently, P1 was used to encapsulate Pt-1 via electrostatic interactions. As shown in Fig. 1A, NP@Pt-1 had a spherical morphology and a comparable hydrodynamic diameter at∼150 nm with a narrow particle size distribution (polydispersity index (PDI): 0.089) (Fig. 1B).

To find out whether the drug encapsulation could change the light responsiveness of Pt-1, UV−vis spectra of Pt-1 and NP@Pt-1 were further recorded. Compared with free Pt-1, NP@Pt-1 showed an 8 nm blue shift and a broader peak width, possibly indicating the aggregation of Pt-1 inside the polymeric core of NP@Pt-1 (Fig. 1C). Furthermore, to visualize and quantify the uptake of nanoparticles by tumor cells, we then investigated the intracellular uptake of NP@Pt-1 via confocal laser scanning microscope (CLSM). CT26 colon cancer cells, a widely used murine cancer cell line of colon adenocarcinoma, were treated with Cy5.5 labelled NP@Pt-1. Subsequently, the cells were visualized via CLSM. As shown in Fig. 1D, the blue color came from the nuclei stained with DAPI, while the red color and green color came from Cy 5.5 labeled NP@Pt-1 and the cytoskeleton stained with Alexa 488, respectively, indicating the distribution of NP@Pt-1 in the cell cytosol. Within 1 h of treatment, NP@Pt-1 was internalized by CT26 cancer cells as shown by the intracellular red fluorescence. Moreover, the red fluorescence was found to be steadily increased within 7 h, which indicated a time-and energy-depended cellular uptake of NP@Pt-1. This was further confirmed by flow cytometry (Fig. 1E, F). As there are Pt atoms in NP@Pt-1, it is possible to quantify the intracellular uptake by ICP-MS. Therefore, CT26 cells treated with Pt-containing drugs at various time points was extracted and the Pt in the cells was tested. As shown in Fig. 1G, after 7 h treatment, the Pt uptake in the CT26 cells increased from 3.8 ng Pt/million cells to 9.5 ng Pt/million cells (Fig. 1G), which was also in accordance with the flow cytometry results, suggesting the time-dependent efficient cellular uptake of the NP@Pt-1. Interestingly, Pt-1 exhibited higher intracellular colocalization than NP@Pt-1 possibly due to the positive charge after chelating with cationic porphyrin, which may result in undesirable toxic effect in vivo [28].

**Fig. 2:**
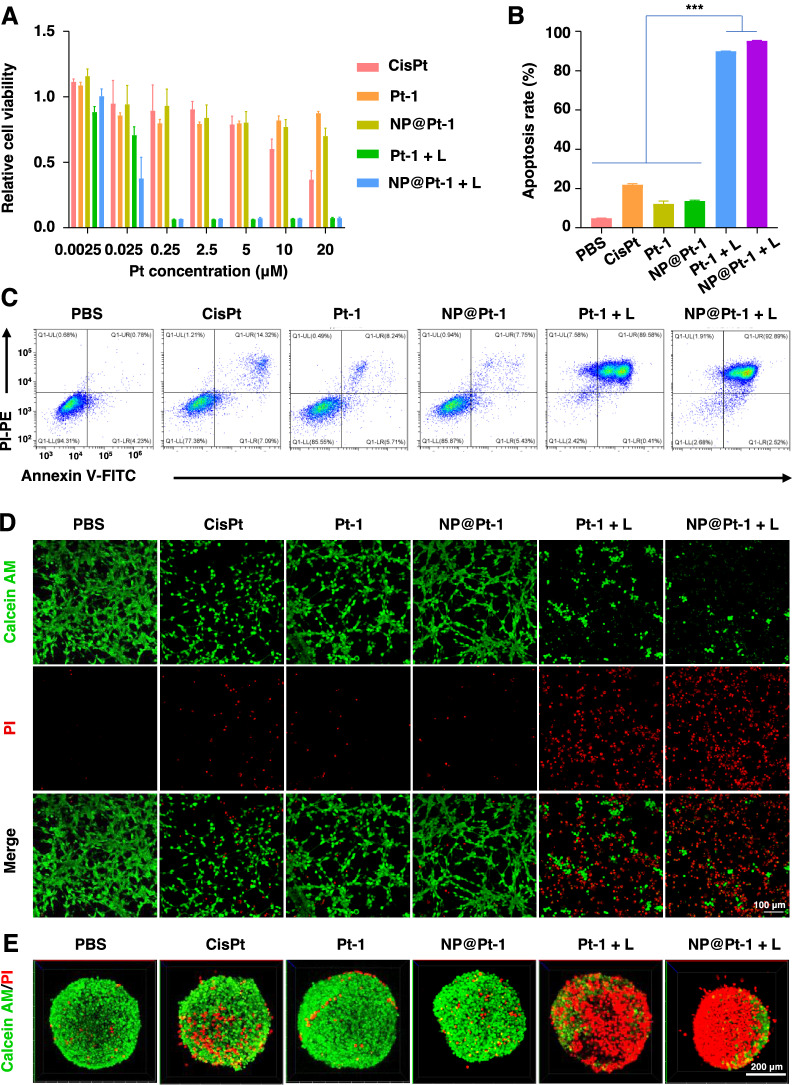
The anticancer activity of NP@Pt-1. **A** Relative cell viability of CT26 cells with various treatments by an MTT assay. **B** Quantification of apoptosis rates of CT26 cells treated with different drugs. **C** Apoptosis rates of CT26 cells by flow cytometry. **D** Calcein/PI staining images of CT26 cells following different treatments. Calcein labeled live cells were shown in green fluorescence, and PI labeled dead cells were shown in red fluorescence. **E** Calcein/PI staining images of CT26 on 3D tumor spheroids following various treatments. (***p < 0.001)

To further investigate the anticancer efficacy of NP@Pt-1 in vitro, the cytotoxicity of the different formulations of NP@Pt-1 was tested via an MTT assay. As shown in Fig. 2A, the viability of CT26 cells remained nearly 80% after incubation with Pt-1 and NP@Pt-1 at a concentration ranging from 0.0025 to 20 μM without light irradiation, while 50% of CT-26 cells were killed at 20 μM of cisplatin, indicating lower toxicity of the Pt-1 itself [26]. Notably, only after 15 min of light irritation (420 nm, 6.95 J cm^−2^), NP@Pt-1 + L (with light irradiation) and Pt-1 + L (with light irradiation) exhibited significantly stronger anticancer efficacy as above 90% of cells were killed. Additionally, the ability of NP@Pt-1 to induce cancer cell apoptosis was studied by an Annexin V-FITC/PI assay. As shown in Fig. 2B and C, the apoptosis rates induced by cisplatin, Pt-1 and NP@Pt-1 were 22.31%, 9.95%, and 13.28%, respectively. In contrast, Pt-1 + L and NP@Pt-1 + L induced significantly augmented apoptosis rate up to 90.09% and 95.41%, respectively, which was clearly in accordance with their anti-cancer activity by the above MTT assay (Fig. 2A). Moreover, CLSM were applied to visualize both the dead and live cells after treatment with various Pt-containing formulations via a calcein-AM and PI double staining live-dead assay (Fig. 2D). Theoretically, live cells could enzymatically hydrolyze the non-fluorescent calcein-AM to the green fluorescent calcein, while PI can penetrate the cell membrane of dead cells and bind to DNA to emit red fluorescence [25]. As shown in Fig. 2D, cells treated with Pt-1 + L and NP@Pt-1 + L showed the highest red fluorescence among all the other groups, indicating the strongest cancer cell killing efficiency of NP@Pt-1 + L. Finally, the toxicity of NP@Pt-1 was further confirmed on a 3D tumor cell spheroid with Calcein-AM /PI staining. As shown in Fig. 2E, the spheroid of the untreated cells were stained green (live cell) by Calcein-AM, while the spheroid of NP@Pt-1 + L had the most red fluorescence dots (dead cell), indicating that synergistic anti-cancer effect generated by ROS and cisplatin-like drugs from NP@Pt-1.

**Fig. 3:**
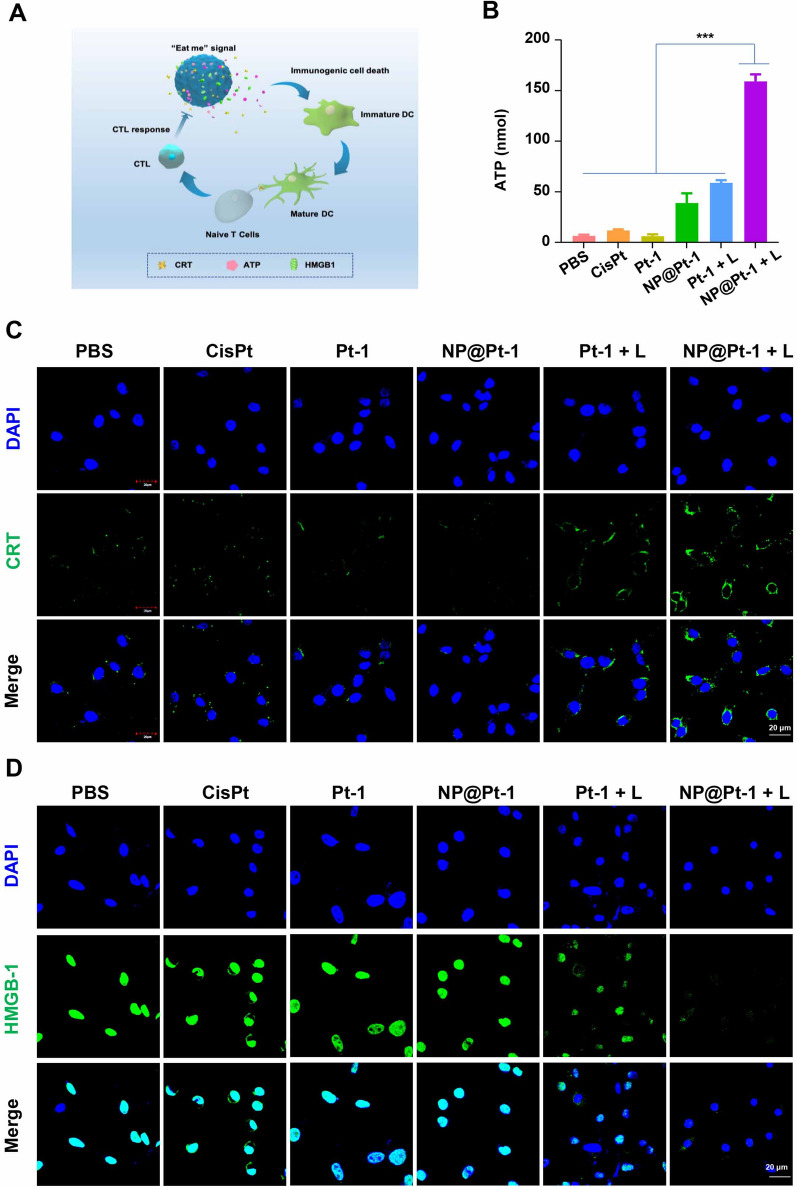
NP@Pt-1 induced ICD in vitro. **A** Schematic illustration of the ICD induced by NP@Pt-1. **B** Extracellular ATP levels in CT26 cells after various treatments. **C** Translocation of CRT to the surface of CT26 cells after various treatments. Scale bar = 20 μm. (n = 3, ***p < 0.001). **D** HMGB1 released from CT26 cells after various treatments. Scale bar = 20 μm

The ICD effect could be characterized by the presence of various DAMPs, such as release ATP and HMGB and translocation of CRT [12] (Fig. 3A). Therefore, to prove this, the release of ATP into the dying CT26 cells treated with NP@Pt-1 was studied by an ATP Assay Kit. After 12 h, cancer cells treated with NP@Pt-1 + L secreted 3 times more ATP than cells treated with Pt-1 + L in vitro (178 nmol versus 47 nmol, respectively) (Fig. 3B). Moreover, NP@Pt-1 + L and Pt-1 + L treatment significantly increased the translocation of CRT and HMGB1 release by CLSM (Fig. 3C, D). To analyze the membrane surface exposed DAMPs, i.e., CRT (ecto-CRT), cells were firstly treated with NP@Pt-1 and then labeled with anti-CRT antibodies and fluorescent secondary antibodies for CLSM observation. As shown in Fig. 3C, NP@Pt-1 + L showed enhanced effects on triggering CRT exposure compared to that of Pt-1 + L.Notably, both HMGB1 release and ATP secretion in cells treated with NP@Pt-1 + L were dramatically enhanced over those treated with Pt-1 + L (Fig. 3D), which was proved by the quantification results of CRT translocation (Fig. 3C) and HMGB1 released (Fig. 3D) from cell culture (Additional file 1: Fig. S4). Taken together, these results indicated that NP@Pt-1 increased DAMPs exposure for ICD effect.

**Fig. 4:**
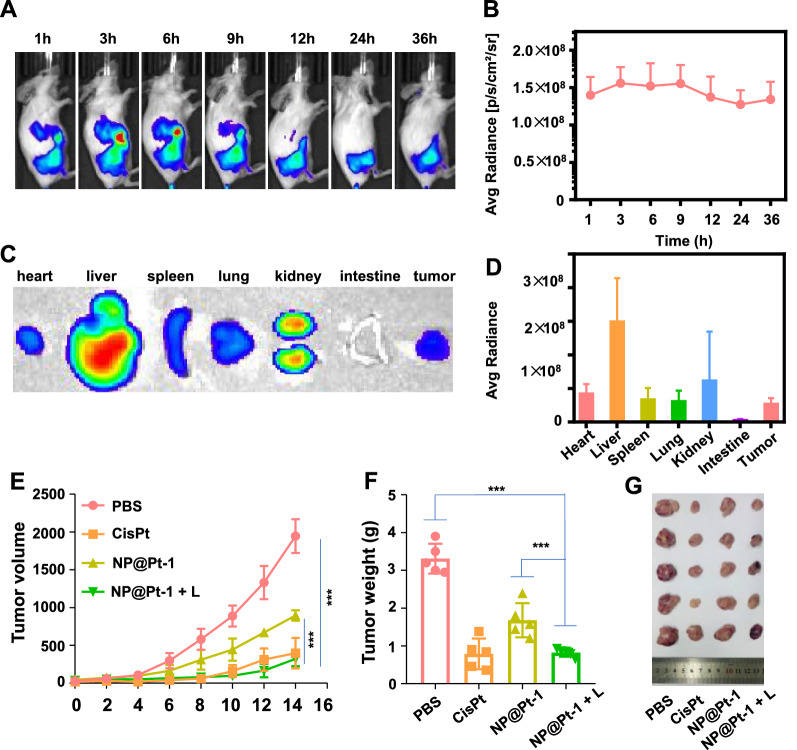
In vivo tumor accumulation and anticancer effect of NP@Pt-1 on a CT26 murine colon cancer model. **A** In vivo biodistribution of Cy7.5 labeled NP@Pt-1 via fluorescence imaging. **B** Quantitative measurement of Cy7.5 labeled NP@Pt-1. **C** Ex-vivo fluorescence imaging of various organs and tumors at 36 h after intravenous administration. **D** Quantitative study of *ex-vivo* fluorescence distribution of various organs and tumors at 36 h after intravenous administration. **E** Schematic illustration of the timeline of in vivo treatment on a CT26 murine colon cancer model. **F** Tumor growth inhibition curves after various treatment. **G** Ex-vivo tumor weight measurement after various treatment. **H** Ex-vivo tumor imaging after various treatment (n = 5, ***p < 0.001).

Next, the anticancer efficacy of NP@Pt-1 in CT-26 tumor-bearing mice were examined. BALB/c mice were subcutaneously injected with CT26 colon carcinoma cells in the flank on day 0 and the intravenous administration of Cy7.5 labelled NP@Pt-1 was performed. Firstly, the noninvasive whole-animal imaging over time was conducted and the results revealed that the mice administered with NP@Pt-1 had the maximized fluorescence signal at tumor sites during the first 3–9 h and the fluorescence remained up to 36 h (Fig. 4A). The average radiance was shown the same (Fig. 4B, defined as fluorescence intensity/area/time). Notably, the ex vivo study of the major organs and tissues was performed and the results showed that after administration of NP@Pt-1 at 36 h, at least eight-fold greater red fluorescence intensity coming from Cy7.5 in tumor tissues was detected than that in the intestine (Fig. 4C, D). Moreover, the red fluorescence of NP@Pt-1 was strong in the liver and kidneys. Secondly, the therapeutic study of NP@Pt-1 and its effect on antitumor immune responses in vivo were examined. BALB/c mice were inoculated subcutaneously with CT26 cells. When the tumor size reached ~ 100 mm^3^, the mice were treated with cisplatin (3.5 mg/kg), NP@Pt-1(3.5 mg/kg) and NP@Pt-1(3.5 mg/kg) + L (420 nm laser irritation). At this dose, NP@Pt-1 was found to have moderate impact on the overall tumor growth, compared with PBS (Fig. 4E–G). In contrast, NP@Pt-1 + L significantly inhibited the tumor growth on a CT-26 mouse model (Fig. 4E–G).

In the ICD cascade, promoting the maturation of DC cells and improving the tumor infiltration of T cells is essential for effective cancer immunotherapy [29]. Therefore, to prove this, the immune cell population in desirable tissues and the spleen was investigated. The DC maturation, T cell proliferation and infiltration in tumor-draining lymph nodes (TDLN), spleen and tumor after various treatments were studied.

**Fig. 5:**
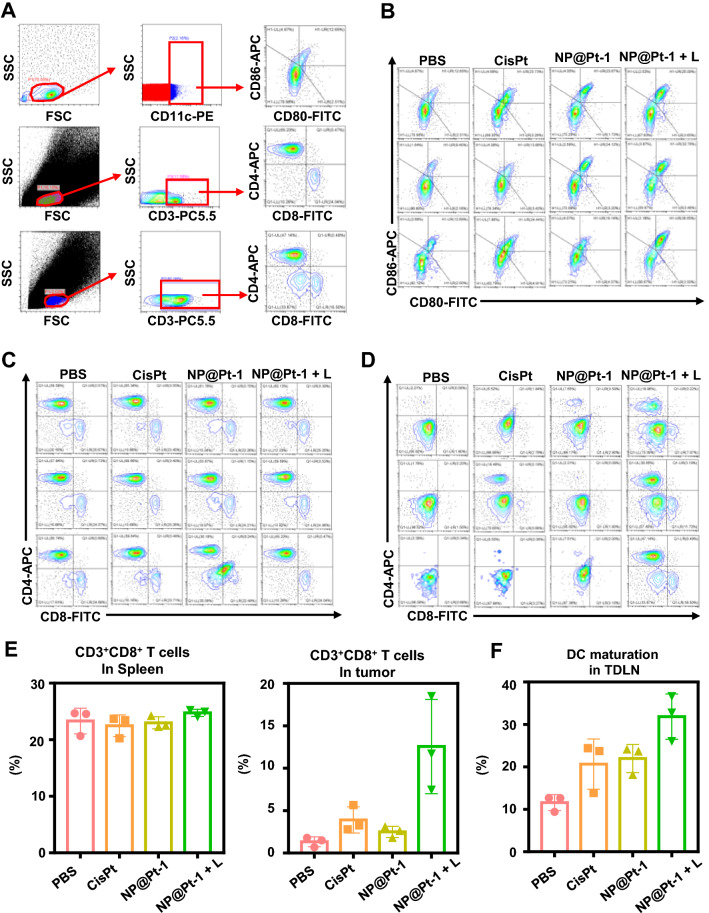
The ICD cascade generated by NP@Pt-1 promoted the cancer  immune response in vivo. **A** Gating strategies for flow cytometric analysis of tumor, peripheral lymphocytes and spleen. **B** Flow cytometry study of DC maturation in tumor-draining lymph nodes. Cells were stained with CD11c antibody. Gating on CD11c^+^ cells, the percentage of DC maturation as indicated by the portion of CD80^+^CD86^+^ cells can be identified. **C**, **D** Flow cytometry study of T cell proliferation in spleen (**C**) and CT26 tumor (**D**). Cells were stained with CD3 antibody. For gating on CD3^+^ cells, we identified the percentage of CD8^+^ T cells as indicated by the portion of CD8^+^ cells. **E** Percentage of CD3^+^CD8^+^ T cell in spleen and CT26 tumor. **F** Percentage of CD80^+^CD86^+^ cells in tumor-draining lymph nodes. Data are shown as means ± SD, n = 3; *p < 0.05, **p < 0.01

To understand the changes in immune cell population within the TME after different treatments, the residual tumors were first collected and analyzed by flow cytometry (Fig. 5). The gating strategies for flow cytometric analysis of tumor, peripheral lymphocytes and spleen was shown in Fig. 5A. Subsequently, the mature DCs in the TDLN (Fig. 5B), the effector killer T cells in spleen and tumors (Fig. 5C, D) were studied, which were further quantified thereafter (Fig. 5B–F). As shown in Fig. 5B and F, the mature DCs (CD11_C_^+^CD80^+^CD86^+^) [29] in the TDLN of mice treated with NP@Pt-1 + L were the highest at 30%, which were much higher than those of mice treated with cisplatin and NP@Pt-1without light irradiation at 20% and 22% respectively. Moreover, NP@Pt-1 + L demonstrated a potent ability to promote DC maturation compared to the other treatment groups, suggesting the necessity of light irritation of NP@Pt-1 for DCs maturation. To evaluate the side effect, the amount of proliferative effector killer T cells (CD3^+^CD8^+^) in the spleen were measured [30, 31]. Notably, the results showed there was no significant increase in the amount of CD8^+^ T cells in the spleen of mice treated with NP@Pt-1 + L (Fig. 5C–E). Previous study indicated that the eliciting of the tumor T cell infiltration via ICD could induce a strong antitumor immune response [18]. Results showed that CD8^+^ T cells in tumor tissues of mice treated with NP@Pt-1 + L were about 3 -fold more than those of all other groups (15% *vs.* 4%) (Fig. 5A, D, E). Therefore, we can conclude that NP@Pt-1 + L significantly increased the proportion of CD8^+^ T cells. Taken together, the above results confirmed that NP@Pt-1 effectively induced ICD cascade and improved the cancer immunotherapy.

## Conclusion

Chemotherapy can enhance the immunogenicity of tumor cells through a variety of mechanisms when it non-specifically kills tumor cells. After the tumor cells die, they are transformed from non-immunogenic to immunogenic which then can mediate anti-tumor immune responses, resulting in a so-called ICD effect. In recent years, numerous researches were carried out on ICD, mainly focusing on chemotherapeutic drugs, radiation therapy and PDT. PDT is a relatively novel non-invasive treatment modality for cancer which relies on specific wavelength light sources to activate photosensitizers in tumor tissues to produce ROS to kill tumors. This work here reported a safe and effective ROS responsive drug delivery system for chemotherapy, PDT and cancer immunotherapy. By delivering a porphyrin-cisplatin conjugate with polymeric nanocarriers, NP@Pt-1 can kill the cancer cells by Pt–DNA binding and PDT mediated ROS generation to damage cell membranes, lipids, proteins and DNAs. More importantly, the generated ROS could induce ICD for immunotherapy. This triple action anticancer approach may be readily applied to other chemotherapeutic agents. Our strategy highlighted the translation of a triple action drug for cancer chemotherapy and immunotherapy.

## Supplementary Information

**Additional file 1: Figure S1.** Synthesis route of Pt-1. **Figure S2.** Synthesis route of P1. **Figure S3.** ^1^H NMR study of Pt-1 in DMSO-d_6_. **Figure S4.** Relative signals quantification of CRT and HMGB-1 for CT26 cells treated by various formulations (n=3, P<0.001).

## Data Availability

All data used to generate these results is available within the paper and the Supporting Information.
