# Supplementary material for: Light triggered release of a triple action porphyrin-cisplatin conjugate evokes stronger immunogenic cell death for chemotherapy, photodynamic therapy and cancer immunotherapy
Source: J Nanobiotechnology. 2022 Jul 16;20:329. doi: 10.1186/s12951-022-01531-5 (PMC9287983; doi:10.1186/s12951-022-01531-5)
Supplement: Supplementary file 1 — Additional file 1: Figure S1. Synthesis route of Pt-1. Figure S2. Synthesis route of P1. Figure S3. 1H NMR study of Pt-1 in DMSO-d6. Figure S4. Relative signals quantification of CRT and HMGB-1 for CT26 cells treated by various formulations (n=3, P<0.001). [file 12951_2022_1531_MOESM1_ESM.docx]

**Additional Information**

**Light Triggered Release of a Triple Action Porphyrin-Cisplatin Conjugate Evokes Stronger Immunogenic Cell Death for Chemotherapy, Photodynamic Therapy and Cancer Immunotherapy**

Haiqin Song^1#^, Zhenghao Cai^1#^, Juyi Li^3^, Haihua Xiao^3*^, Ruogu Qi^4^*, Minhua Zheng^1^*

^1^ Department of General Surgery, Ruijin Hospital, Shanghai Jiaotong University, School of Medicine, Shanghai,20023 China

^2^ Department of Materials Science and Chemical Engineering，Stony Brook University, Stony Brook, NY, 11794

^3^ Beijing National Laboratory for Molecular Sciences, State Key Laboratory of Polymer Physics and Chemistry, Institute of Chemistry, Chinese Academy of Sciences, Beijing 100190, China

^4^ School of Medicine & Holistic Integrative Medicine, Nanjing University of Chinese Medicine, Nanjing, 210023, China

^#^ These authors contribute equally

^*^ E-mail address:

zmhtiger@yeah.net (M. Zheng),

rqi@njucm.edu.cn (R. Qi),

hhxiao@iccas.ac.cn (H. Xiao).

**Materials & Methods**

**Materials**

5,10,15,20-Tetra(4-pyridyl)-21H,23H-porphine, mercaptoacetic acid, lithium aluminum hydride (LiAlH_4_), polyethylene glycol monomethyl ether (mPEG_5k_-OH, average molecular weight 5,000), 3-(4,5-dimethylthiazol-2-yl) -2,5-diphenyltetrazolium bromide (MTT), and sodium dodecyl sulfate (SDS) were purchased from Aladdin, Shanghai. 1,2,4,5-cyclohexanetetracarboxylic dianhydride (HPMDA), were purchased from Energy-chemical, Shanghai. 2-(4-amidinophenyl)-1H-indole-6-carboxamidine (DAPI), Cy5.5, Cy7.5, and FITC Phalloidin were purchased from Solarbio, Beijing. Annexin V-FITC/PI Cell Apoptosis Kit and Calcein/PI Live/Dead Viability/Cytotoxicity Assay Kit were purchased from Beyotime Biotechnology, Shanghai.

**Cell lines and animals**

The RPMI-1640 and Modified Eagle’s Medium (DMEM) medium was mixed with 10% fetal bovine serum (FBS) and 1% antibiotics (penicillin and streptomycin) for the culture of CT-26 cells. The culture condition is 37 °C in a humidified environment containing 5% CO_2_. For cell harvesting, 0.5% w/v trypsin in phosphate-buffered saline (PBS) was used to detach from the cell culture dish, and then resuspended in fresh medium for the following usage.

Balb/c mice (female, 4 weeks old) were purchased from Vital River Laboratory Animal Technology Co. Ltd. (Beijing, China) and raised in SPF animal rooms. All animal experiments were conducted under guidelines evaluated and approved by Peking University Institutional Animal Care and Use Committee (LA2021316).

**Synthesis of Pt-1**

Cisplatin (CisPt) (0.25 mmol, 75 mg) and silver nitrate (0.25 mmol, 43 mg) were dissolved in 5 mL of N,N-Dimethylformamide (DMF) and stirred at room temperature for 24 h. The resulted turbid solution was centrifuged to remove the white silver chloride. The light-yellow colored solution was added to a suspension of 5,10,15,20-Tetra(4-pyridyl)-21H,23H-porphine (Porphyrin) (0.0625 mmol, 39 mg) in 5 mL DMF and stirred at 80 °C for 48 h in the dark. After that, the reaction mixture was cooled down to room temperature and precipitated with diethyl ether. The solid was filtered and further washed with methanol, dichloromethane and diethyl ether. The solid was further dried in vacuum as Pt-1 [1]. ^1^H NMR (400 MHz; DMSO-d_6_): δ 9.22(o-pyridyl, d, 8H, J = 6.5 Hz), 9.02 (β-pyrrole, s, 8H), 8.46 (m-pyridyl, d, 8H, J = 6.5 Hz), 4.95 (NH_3_, s, 12H), 4.49 (NH_3_, s, 12H), -3.08 (NH, s, 2H).

**Synthesis of 2,2'-(propane-2,2-diylbis(sulfanediyl)) bis(ethan-1-o1) (PSDE)**

Mercaptoacetic acid (16 mL) and acetone (40 mL) were placed in a three-necked bottle followed with continuous injection of dry HCl. The mixture was stirred at room temperature for 2 h. Thereafter, the reaction solution was filtered to obtain the light-yellow solid, which was further washed with ethyl acetate and water, respectively. The white product (2,2'-(propane-2,2-diylbis(sulfanediyl))diacetic acid, PDSDA) was then obtained and dried under vacuum for next steps.

The above-synthesized PDSDA (5 g) was suspended in anhydrous tetrahydrofuran (THF), cooled in an ice bath for 15 min. Lithium aluminum hydride (2.5 g) was then slowly added in the mixture and heated to 50℃ for 8 h, which was subsequently quenched by saturated sodium hydroxide solution. The organic phase of the above solution was separated by ethyl acetate extraction and dried by anhydrous magnesium sulfate. The mixture was further filtered and steamed, and finally a yellow viscous product namely PSDE was obtained *via* column chromatography as previously described [2].

**Synthesis of P1**

PSDE (0.1 mmol) and 1,2,4,5-cyclohexanetetracarboxylic dianhydride (HPMDA) (0.11 mmol) were placed in a 50 mL round-bottom flask. Subsequently,10 mL DMF was added into the flask under continuous stirring for 48 h. Subsequently, mPEG_5k_-OH (0.02 mmol) was added for end-capping of the polymer for 24 h. The final product poly (PSDE-co-HPMDA)-mPEG (P1) was collected *via* dialysis and dried under vacuum.

**Preparation and characterization of NP@Pt-1**

The as-synthesized polymer P1 (10 mg) and Pt-1 (1 mg) were initially dissolved in 1 mL DMF, and then the solution was dispersed in 10 mL de-ionized water under continuous stirring. After vigorously stirring for 15 min, the mixture was collected and dialyzed with a dialysis bag (MWCO: 3500 Da) overnight. The nanoparticles (NP@Pt-1) were then separated by centrifugation and washed twice with de-ionized water.

The morphology of NP@Pt-1 was characterized by TEM (HT-7700, Hitachi, Japan). The size of NP@Pt-1 was characterized by Malvern Zetasizer Nano ZS90 laser particle size analyzer (Nano ZS, UK). The UV-vis absorption spectra of free Porphyrin, Pt-1 and NP@Pt-1 were measured by UV/vis spectrometry (UV-1800, Mapada, Shanghai). Pt-1 in NP@Pt-1 was quantitatively determined by UV absorption.

**In *vitro* cellular uptake of NP@Pt-1 by CLSM and** **flow cytometry.**

The as-synthesized polymer P1 (10 mg), Pt-1 (1 mg), and Cy5.5 (0.1 mg) were initially dissolved in 1 mL DMF, and then the solution was dispersed in 10 mL de-ionized water with continuous stirring. After vigorously stirring for 15 min, the mixture was collected and dialyzed using a dialysis bag (MWCO: 3500 Da) overnight. The nanoparticles (NP@Pt-1@Cy5.5) were then separated by centrifugation and washed twice with de-ionized water.

The intracellular uptake of NP@Pt-1@Cy5.5 was analyzed by CLSM and flow cytometry. For CLSM (OLYMPUS FV1000-IX81, Olympus, Japan) observation, CT26 cells were seeded into 24-well chambered slides (Thermo Scientific, USA) at a density of 2 × 10^4^ cells per well and incubated with RPMI1640 supplemented with 10% FBS (1 mL) at 37 °C for 12 h. After removing the medium, the cells were treated with NP@Pt-1@Cy5.5 at an equivalent Cy5.5 concentration (10 μg/mL) for 0.5 h, 3 h, and 6 h. Then the medium was removed and were subsequently incubated with FITC Phalloidin according to the manufacturer’s protocol [2]. Subsequently, the cells were stained with DAPI, and then observed by CLSM. For flow cytometry, CT26 cells were seeded into 12-well plates (20 × 10^4^ cells/well) and cultured for 12 h. After treatment with NP@Pt-1@Cy5.5 at an equivalent Cy5.5 concentration (10 μg/mL) for 0.5 h, 3 h, and 6 h, the cells were collected and analyzed *via* flow cytometry (Becton Dickinson and Company, USA).

**Platinum uptake**

To determine the cellular uptake, CT26 cells were seeded in 6-well plates with a density of 1×10^6^ cells per well overnight. The cells were then treated with CisPt, Pt-1 and NP@Pt-1 at a Pt concentration of10 μM. After incubation at 37 °C for 1 h, 4 h, and 7 h respectively, the cells were then washed with PBS for three times and lysed by cell lysis buffer. The platinum content in the cell lysis solution were determined by ICP-MS.

**Cell viability**

An MTT assay was utilized to examine the cytotoxicity. CT26 cells were seeded in 96-well plates (Thermo Scientific, USA) at a density of 8,000 cells per well and cultured for 12 h. Cells were incubated with CisPt, Pt-1 and NP@Pt-1 at a Pt concentration ranging from 0.005, 0.05, 0.5, 5, 10, 20, 40 µM. The cells were incubated with the previous drugs for 6 h, and the medium was then replaced by fresh medium without drugs. The plates were then irradiated 15 minutes at 420 nm (6.95 J/cm^2^) in a Rayonet Chamber Reactor respectively. Upon further incubation for 12 h, the medium was removed, and 10% MTT diluted with RPMI1640 (100 μL) was added in the wells. After incubation in 37 °C for 4 h, 10% SDS (100 μL) was added to each well and incubated at 37 °C for another 12 h. The absorbance of wells was read by a Microplate reader (SpectraMax) at 570 nm (peak absorbance) and at 650 nm (background absorbance). The cell viability was expressed as the ratio of the absorbance of the test wells and control wells, and data are shown as the mean ± standard deviation (S.D.).

**Cell apoptosis study**

CT26 cells were seeded in 6-well plates (Thermo Scientific, USA) at a density of 2 × 10^5^ cells per well and cultured for 12 h. Cells were then incubated with CisPt, Pt-1 and NP@Pt-1 at a final Pt concentration of 10 µM for 6 h, the medium was then replaced by fresh medium. The plates were irradiated 15 minutes at 420 nm (6.95 J/cm^2^) in a Rayonet Chamber Reactor. After being incubated for 12 h, the cells were collected and stained by an Annexin V-FITC/PI Cell Apoptosis Kit following the manufacturer’s instruction [2]. The cells were finally collected and analyzed *via* flow cytometry (Becton Dickinson and Company, USA). Moreover, the cells were stained by Live/Dead Viability/Cytotoxicity Assay Kit, according to the manufacturer’s protocol^[3]^, and then observed by laser confocal microscope (OLYMPUS FV1000-IX81, Olympus, Japan). The apoptosis rate of cells was quantified by measuring the signal intensity of related dye against total nucleus.

**Cell cytotoxicity on 3D tumor spheroids**

To culture CT26 cells as 3D spheroids, pre-treated ultra-low attachment plates (Corning) were used. Methylcellulose (0.75%) was added in RPMI 1640 growth medium to prevent excessive aggregation of cells in spheroid culture and to maintain an even spheroid size. The 3D spheroids were then treated with CisPt, Pt-1 and NP@Pt-1 at a final Pt concentration of 10 µM for 6 h. The medium was then replaced by fresh medium. The plates were then irradiated 15 minutes at 420 nm (6.95 J/cm^2^) in a Rayonet Chamber Reactor respectively. Upon further incubation for 12 h, the 3D tumor spheroids were then stained by a Calcein/PI Live/Dead Viability/Cytotoxicity Assay Kit according to the manufacturer’s protocol [3], and then the cells were observed by CLSM (LSM-800, ZEISS, Germany).

**Detection of ICD biomarkers**

For analyzing surface-exposed CRT, CT26 cells were seeded into 24-well chambered slides (1 × 10^4^ cells/well) and cultured for 12 h. The cells were then treated with CisPt, Pt-1 and NP@Pt-1 at a final Pt concentration of 10 µM for 6 h. The culture medium was then replaced by fresh medium. The plates were then irradiated for 15 minutes at 420 nm (6.95 J/cm^2^) in a Rayonet Chamber Reactor. Upon further incubation for 6 h, the cells were then incubated with anti-calreticulin antibody (Abcam) for 2 h at 4 °C. Subsequently, the cells were incubated with Alexa Fluor 488-conjugated secondary antibody (Abcam) for 1 h. After further stained with DAPI, the cells were observed under CLSM. For flow cytometry analysis, CT26 cells were collected following a similar process described above. Subsequently, the cells were analyzed *via* flow cytometry.

For the extracellular released HMGB1 analysis, CT26 cells were seeded into 24-well chambered slides (1 × 10^4^ cells/well) and cultured for 12 h. The cells were treated with CisPt, Pt-1 and NP@Pt-1 at a final Pt concentration of 10 µM for 6 h. The culture medium was then replaced by fresh medium. The plates were then irradiated for 15 minutes at 420 nm (6.95 J/cm^2^) in a Rayonet Chamber Reactor. Upon further incubation for 12 h, the cells were incubated with anti-HMGB1 antibody (Abcam) for 2h at 4 °C. Subsequently, the cells were incubated with an Alexa Fluor 488-conjugated secondary antibody (Abcam) for 1 h. After staining with DAPI, the cells were observed under CLSM. The quantity of the CRT/HMGB1 expression was quantified by measuring the signal intensity of related dye against total nucleus.

The extracellular released ATP was examined using an ATP Assay Kit**.** Briefly, CT-26 cells were seeded into 24-well plates (5 × 10^4^ cells/well) and cultured for 12 h. The cells were treated with CisPt, Pt-1 and NP@Pt-1 at a final Pt concentration of 10 µM for 6 h. The culture medium was then replaced by fresh medium. The plates were then irradiated for 15 minutes at 420 nm (6.95 J/cm^2^) in a Rayonet Chamber Reactor. Upon further incubation for 12 h, the release of ATP in the cell supernatant was detected by an ATP Assay Kit following the manufacturer’s protocols [4].

***In vivo* imaging and biodistribution**

The polymer P1 (10 mg), Pt-1 (1 mg), and Cy7.5 (0.1 mg) were initially dissolved in 1 mL DMF, and then the solution was dispersed in 10 mL de-ionized water with continuous stirring. After vigorously stirring for 15 min, the mixture was collected and dialyzed using a dialysis bag (MWCO: 3500 Da) overnight. The nanoparticles (NP@Pt-1@Cy7.5) were then separated by centrifugation and washed twice with de-ionized water.

CT26 cells (1 × 10^6^) were subcutaneously injected into the right hip of female BALB/c mice. When the tumor volumes reached about 100 mm^3^, mice were *i.v.* injected with NP@Pt-1@Cy7.5. After drug injection, the FL signals were recorded on IVIS spectrum imaging system (Spectrum CT, PerkinElmer, Ex/Em=740 nm/800 nm) at 1, 3, 6, 9, 12, 24, and 36 h respectively. For the biodistribution study, the mice were sacrificed after 36 h post injection, and the tumors and normal organs were harvested and imaged.

**Establishment of a CT26 tumor model for therapeutic effect study**

CT26 cells (1 × 10^6^) were subcutaneously injected into the right hip of female BALB/c mice. When the tumor volume reached about 100 mm^3^, the mice were *i.v.* injected with CisPt and NP@Pt-1 at a dose of 3.5 mg Pt kg^−1^, respectively. At 24 h and 48 h after injection, the mice treated with NP@Pt-1 were irradiated 15 minutes at 420 nm (6.95 J/cm^2^) in a Rayonet Chamber Reactor respectively. The tumor volume was recorded every other day. The H&E assays of tumor samples were performed to detect apoptotic cells. The tumor tissues sections were further stained by an H&E assay kit. Images were captured by CLSM.

**ICD effect *in vivo***

Mice bearing CT26 tumors were randomly divided into 4 groups (3 mice in each group) when the tumor size reached ~100 mm^3^. The mice were then given PBS, CisPt, NP@Pt-1 and NP@Pt-1 + L. After 2 days of treatment, the tumor, spleen and lymph were collected, which were then treated with Matrigel, and ground through a 70 μm filter. The samples were then resuspended and blocked with 0.1% BSA in PBS, and stained with the corresponding antibody prepared in 0.1% BSA in PBS for 1 h at room temperature for flow cytometry study (FCM, Beckman Coulter, U.S.A.).

**Statistical Analysis.**

The data shown were presented as mean ± standard deviation. Student’s *t*-test was used to determine the statistical difference between various groups. Differences were considered statistically significant at a level of *p < 0.05; **p < 0.01; ***p < 0.001.

**Figure S1.** Synthesis route of Pt-1.

**Figure S2.** Synthesis route of P1.


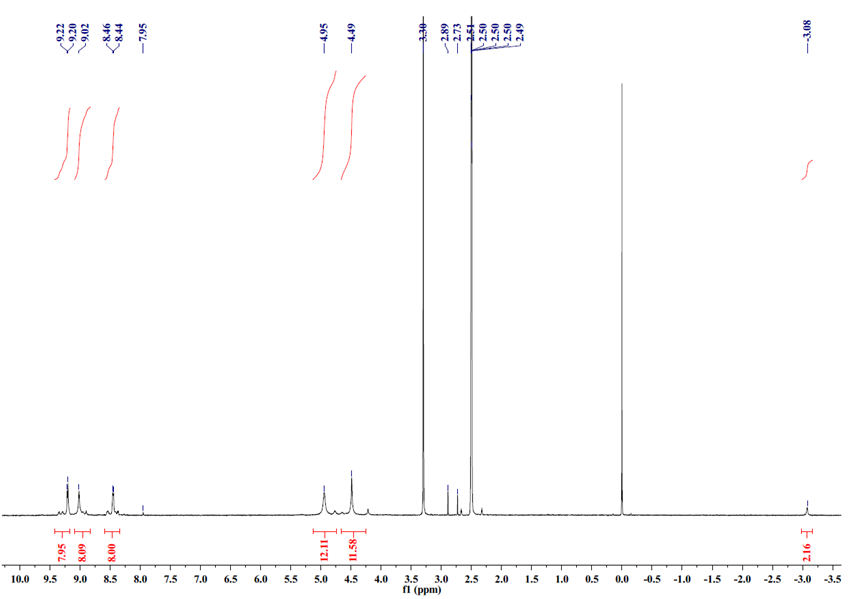


**Figure S3.** ^1^H NMR study of Pt-1 in DMSO-d_6_.

**Figure S4.** Relative signals quantification of CRT and HMGB-1 for CT26 cells treated by various formulations (n=3, P<0.001).

**References**

[1] A. Naik, R. Rubbiani, G. Gasser, B. Spingler. Visible-light-induced annihilation of tumor cells with platinum-porphyrin conjugates. Angew Chem Int Ed Engl 2014, 53, 6938-6941.

[2] F. Ding, L. Zhang , H. Chen, H. Song, S. Chen, H. Xiao. Enhancing the chemotherapeutic efficacy of platinum prodrug nanoparticles and inhibiting cancer metastasis by targeting iron homeostasis. Nanoscale Horiz. 2020;5:999-1015..

[3] Y. Yu, D. Tang, C. Liu, Q. Zhang, L. Tang, Y. Lu, H. Xiao. Biodegradable Polymer with Effective Near-Infrared-II Absorption as a Photothermal Agent for Deep Tumor Therapy. Adv Mater 2022, 34, e2105976.

[4] F. Ding, F. Li, D. Tang, B. Wang, J. Liu, X. Mao, J. Yin, H. Xiao, J. Wang, Z. Liu. Restoration of the Immunogenicity of Tumor Cells for Enhanced Cancer Therapy via Nanoparticle-Mediated Copper Chaperone Inhibition. Angew Chem Int Ed Engl 2022, DOI: 10.1002/anie.202203546.
